# Identification of long non-coding RNA signature for paclitaxel-resistant patients with advanced ovarian cancer

**DOI:** 10.18632/oncotarget.19828

**Published:** 2017-08-02

**Authors:** Luqing Wang, Yanjun Hu, Xiaohong Xiang, Kai Qu, Yue Teng

**Affiliations:** ^1^ Department of Nuclear Medicine, Liaocheng People's Hospital, Taishan Medical College, Liaocheng 252000, China; ^2^ Department of Clinical Laboratory, Liaocheng People's Hospital, Taishan Medical College, Liaocheng 252000, China; ^3^ Department of Hepatobiliary Surgery, The First Affiliated Hospital of Xi'an Jiaotong University, Xi'an 710061, China; ^4^ Department of Obstetrics and Gynecology, The First Affiliated Hospital of Xi'an Jiaotong University, Xi'an 710061, China

**Keywords:** long non-coding RNA, epithelial ovarian cancer, paclitaxel, chemoresistance

## Abstract

Ovarian cancer is the most lethal gynecologic malignancy, characterized by late diagnosis, frequent relapse, and easy development of chemoresistance. Recent studies suggest that lncRNAs are involved in ovarian cancer onset and progression, as well as the resistance in paclitaxel-containing chemotherapy. However, the genome-wide expression pattern and associated functional implications of lncRNAs in paclitaxel-resistant ovarian cancer cells remain undetermined. In the present study, we identified a panel of lncRNAs aberrantly expressed in both paclitaxel resistant ovarian cancer tissues and cell lines, including XR_948297, XR_947831, XR_938728, XR_938392, NR_103801, NR_073113, and NR_036503. Moreover, the seven-lncRNA signature showed a relatively high predictive accuracy of chemoresistance with an area under the ROC curve (AUC) of 0.93, and was associated with progression-free survival inovarian cancer patients (HR=2.05, *p*=0.015). Our function prediction demonstrated that the seven-lncRNA signature was positively correlated with a cluster containing 129 genes enriched in insulin secretion-related pathway. Our findings suggest that the seven-lncRNA signature may be utilized as potent biomarkers for predicting chemoresistance for ovarian cancer patients with paclitaxel-containing chemotherapy.

## INTRODUCTION

Epithelial ovarian cancer (EOC), accounting for the first place in mortality rate of all gynecologic cancers [1], still has a poor prognosis, with a 5-year survival rate lower than 30%, despite the amelioration of modern therapeutic methods [2]. The high mortality rate of EOC is mainly on account of the difficulty in early diagnosis, metastasis, and chemoresistance [3]. Combination of cytoreductive surgery and post-operational chemotherapy is thecurrent standard treatment for advanced EOC. However, more than 70% patients exhibit chemoresistance after surgery, leading to adverse effects to subsequent curative results and survival [4]. The mechanic research on chemoresistance remains clinically challenging, simply because it holds back a successful long-term efficacy of EOC [5, 6]. Currently, the paclitaxel and platinum-containing chemotherapy is the main scheme for dealing with advanced EOC. Compared with platinum resistance which has been widely studied in EOC, much less mechanism of paclitaxel resistance has been clarified. Efforts in exploring molecular mechanism of paclitaxel resistance will definitely enhance the clinical treatment as well as improve the survival rate of EOC patients.

Non-coding RNAs are drawing increasing attention in recent years for their pivotal regulating functions in various biological and pathological processes. As an important member of the non-coding RNAs, long non-coding RNAs (lncRNAs) have been identified as versatile players in multiple diseases by regulating target genes expression, directly either on transcripts originating from the same locus as the lncRNA itself (cis-acting) or on target transcripts originating on other loci (trans-acting) [7]. Numerous evidences reveal that they are significant regulators of various biological processes including immunoreaction, tumorigenesis, genetic modification, and metabolism regulation [8–11], and play pivotal roles in various cellular functions, disease occurrence and progression, including drug resistance [12]. To date, several lncRNAs have been found to mediate drug resistance. For example, studies revealed the harmful effects of a specific lncRNA, named homeobox transcript anti-sense RNA (HOTAIR), with an increasing prevalence of multiple neoplasm chemoresistance phenotypes [13–15]. Huang et al. identified that certain lncRNA could inhibit the transcription of tumor-suppressor genes by regulating protein activity to promote drug resistance in tumor cells [16]. In addition, an eight-lncRNA signature was found to be eligible for classifying patients with poor and improved overall survival rate [17]. Noticeably, two lncRNAs, RP11-284N8.3.1 and AC104699.1.1, have been recently demonstrated to be potential prognosis indicators of EOC patients by lncRNA-mRNA co-regulation network [18]. Collectively, these results confirmed the involvement of lncRNAs in the occurrence and progression of cancer and raised the possibility that lncRNAs could be used as biomarkers for diagnosis and prognosis as well as novel treatment targets [19, 20]. However, so far there is no research available on whether lncRNAs play a role in paclitaxel resistance of EOC. We believe that identifying a paclitaxel resistance-specific signature of lncRNAs in EOC and elaborating the latent molecular mechanisms will broaden our understanding of human EOC and provide future clinical approaches to treating this disease.

The present study aims to demonstrate whether lncRNAs play a vital role in paclitaxel-containing chemotherapies of EOC patients and to assess the prognostic values of identified lncRNAs. Furthermore, we also attempt to explore the underlying molecular mechanisms and involved pathways in paclitaxel resistance of EOC patients.

## RESULTS

### Identification of differentially expressed lncRNAs from the datasets

**Figure 1:**
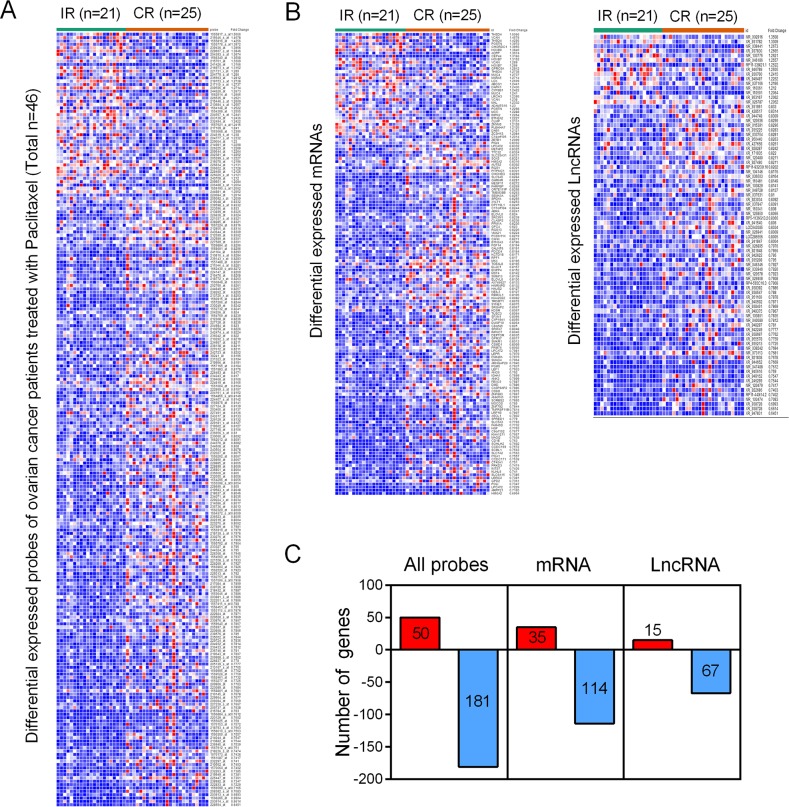
Identification of differentially expressed genes from the EOC dataset GSE30161 **(A)** A total of 231 differentially expressed genes were picked up from EOC patients treated with paclitaxel. **(B)** Within the 231 differentially expressed genes, there were both mRNAs (35 up-regulated mRNAs and 114 down-regulated mRNAs) and lncRNAs (15 up-regulated lncRNAs and 67 down-regulated lncRNAs). **(C)** A diagrammatic sketch demonstrating the specific pattern of differentially expressed genes from GSE30161. IR: incomplete response; CR: complete response.

**Figure 2:**
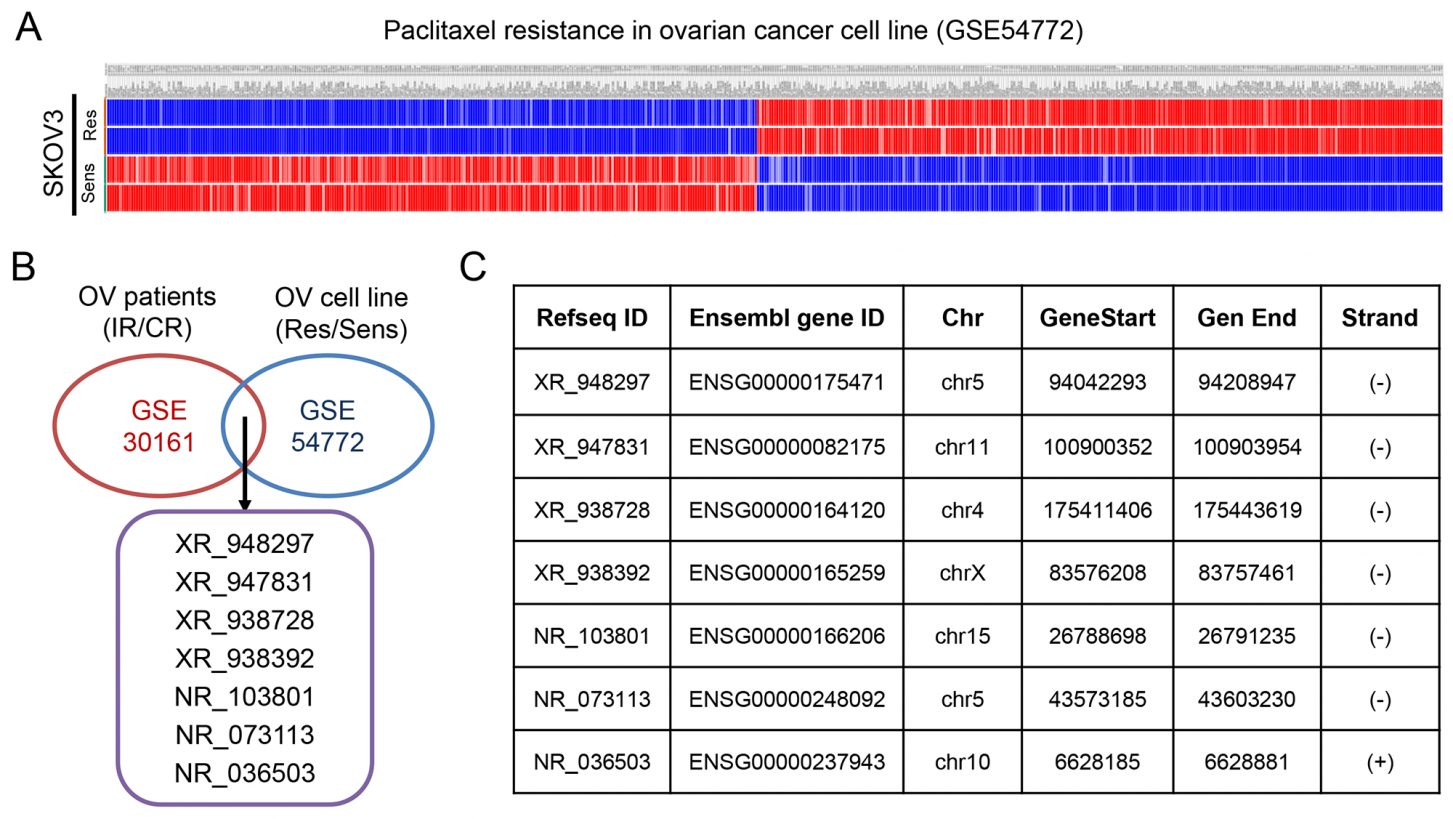
Identification of differentially expressed genes from the EOC dataset GSE54772 **(A)** Approximately 400 differentially expressed genes were picked up from paclitaxel-sensitive and paclitaxel-resistant EOC cell lines. **(B)** A panel of seven lncRNAs(XR_948297, XR_947831, XR_938728, XR_938392, NR_103801, NR_073113, and NR_036503) that were significantly correlated with EOC patients’ chemotherapeutic sensitivity and efficacy were picked up when EOC dataset GSE30161 and GSE54772 were analyzed simultaneously. **(C)** Distribution patterns of the seven enrolled lncRNAs. OV: ovarian cancer; IR: incomplete response; CR: complete response; Res: resistant; Sens: sensitive.

The GSE30161, a gene expression profile consisting of 46 patient samples, was used for the detection of lncRNAs related to incomplete response (IR) and complete response (CR) of EOC patients. With analyzing package GEO2R, we identified a total of 50 up-regulated genes and 181 down-regulated genes (*p*<0.05 and FC>1.2) (Figure 1A), including both mRNAs (35 up-regulated mRNAs and 114 down-regulated mRNAs) and lncRNAs (15 up-regulated lncRNAs and 67 down-regulated lncRNAs) from GSE30161 (Figure 1B and 1C). Then, we analyzed GSE54772, another gene expression profile consisting of samples of 2 paclitaxel-resistant ovarian cancer cell lines as well as 2 paclitaxel-sensitive ovarian cancer cell lines, and found approximately 400 differentially expressed genes between the two groups (*p*<0.05 and FC>2 were considered statistically significant) (Figure 2A). We further analyzed the combination of GSE30161 and GSE54772 and identified a panel of seven lncRNAs(XR_948297, XR_947831, XR_938728, XR_938392, NR_103801, NR_073113, and NR_036503) that were significantly correlated with EOC patients’ chemotherapeutic sensitivity and efficacy (Figure 2B). Distribution patterns of the seven enrolled lncRNAs in different sample groups were presented (Figure 2C).

### Risk assessment and predictive value

**Figure 3:**
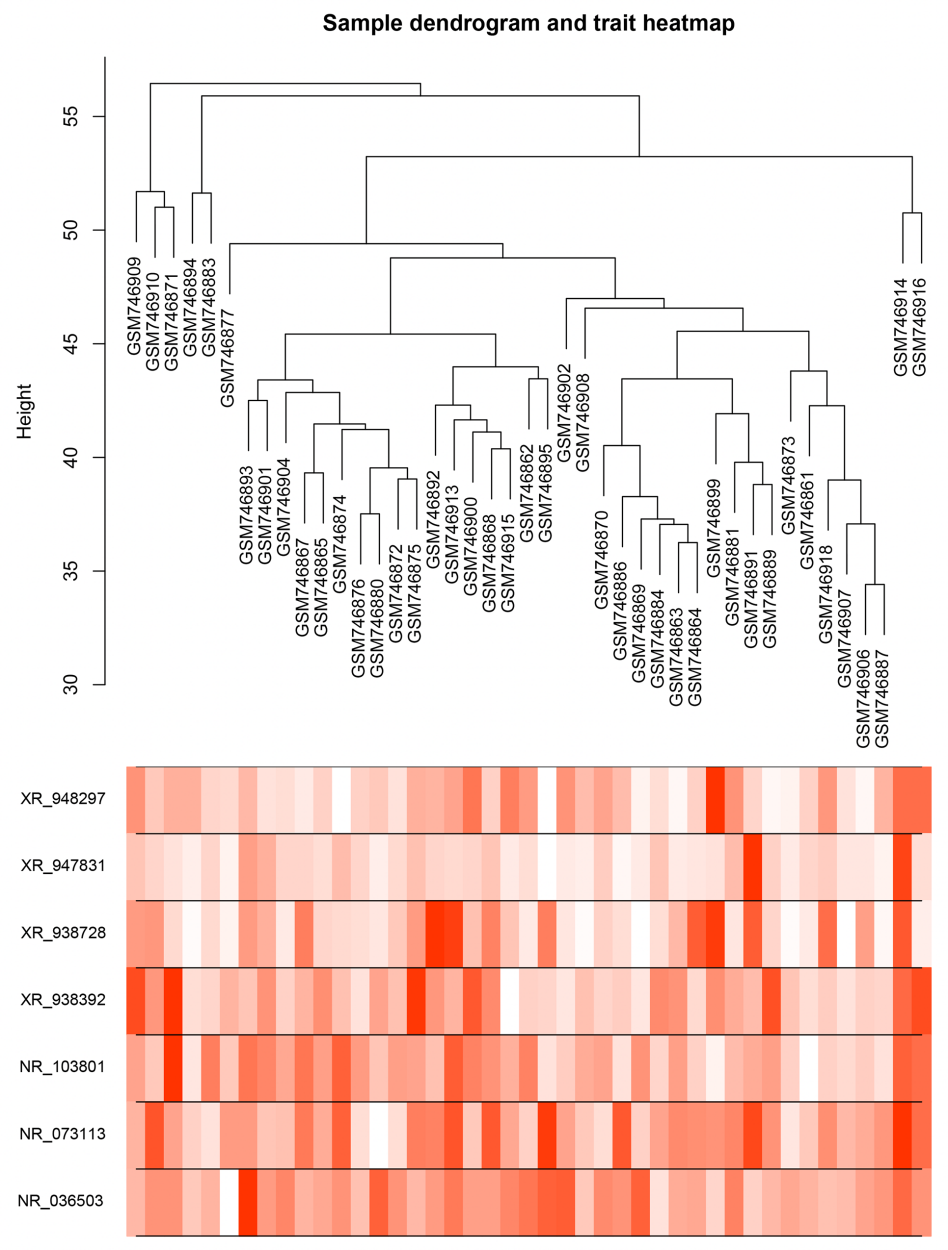
Dendrogram and trait heatmap of seven enrolled lncRNAs between IR and CR EOC patient groups Risk scores were assessed on the basis of the expression levels of seven enrolled lncRNAs between IR and CR EOC patient groups.

**Figure 4:**
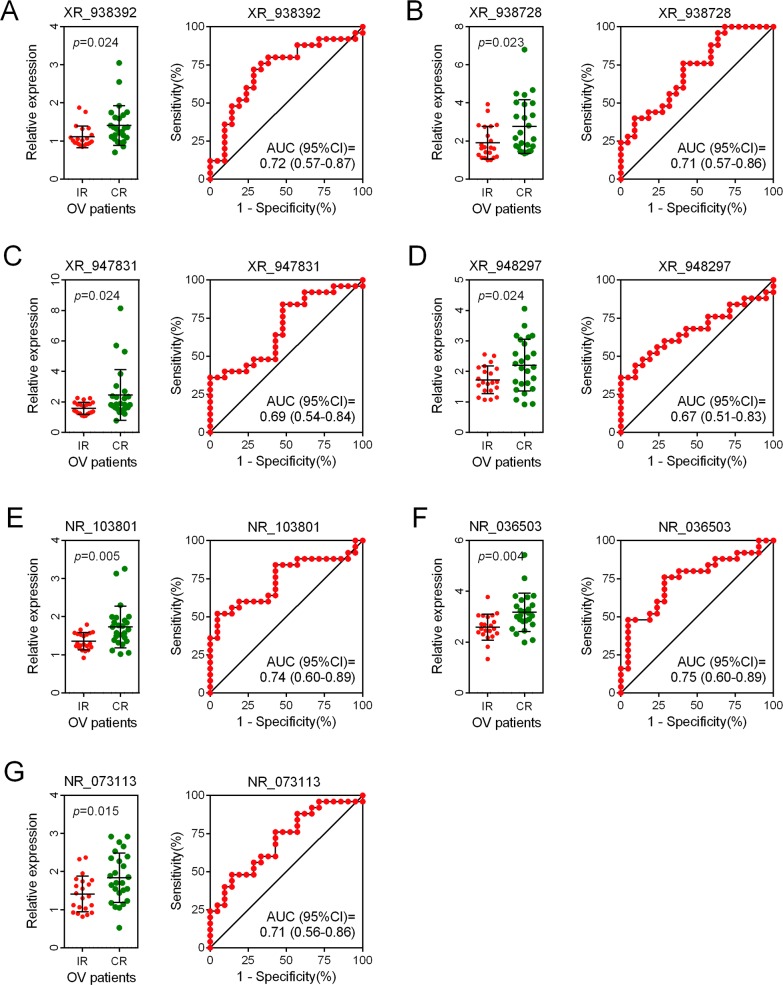
ROC analysis of the predictive accuracy of each individual lncRNA **(A-G)** The predictive accuracy of each individual lncRNA of the seven enrolled lncRNAs was assessed by ROC analysis, respectively. OV: ovarian cancer.

**Figure 5:**
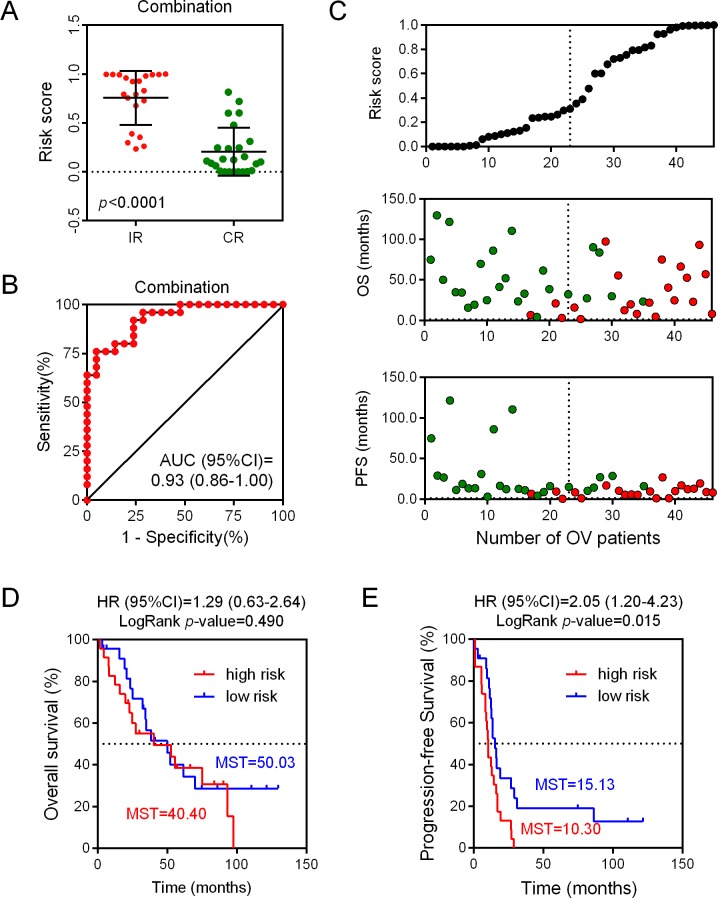
Predictive and prognostic value of each individual lncRNA as well as the combined seven-lncRNA signature **(A)** Risk scores of the combined seven-lncRNA signature between IR and CR EOC patient groups. **(B)** Sensitivity analysis of the combined seven-lncRNA signature between IR and CR EOC patient groups. **(C)** Correlation between risk scores and progression-free survival, as well as overall survival. **(D)** The Kaplan-Meier plotter analysis of overall survival between low risk and high risk EOC patient groups on the basis of the combined seven-lncRNA signature. **(E)** The Kaplan-Meier plotter analysis of progression-free survival between low risk and high risk EOC patient groups on the basis of the combined seven-lncRNA signature. IR: incomplete response; CR: complete response; OS: overall survival; OV: ovarian cancer; PFS: progression-free survival; MST: mean survival time.

We assessed the risk score on the basis of the expression levels of seven enrolled lncRNAs between the two patient groups (IR vs. CR) (Figure 3). Besides the relevance of sensitivity to EOC chemotherapy, the relationships between the identified seven lncRNAs and chemotherapeutic effects were also explored. ROC analysis was performed to assess the predictive accuracy of each individual lncRNA, respectively (Figure 4). Furthermore, we also analyzed the predictive accuracy of the combined seven-lncRNA signature. The seven lncRNAs were integrated into risk score to predict the risk of tumor recurrence of EOC patients receiving paclitaxel-containing chemotherapy. The IR group showed a higher risk score than the CR group (*p*<0.0001) (Figure 5A). Compared with each individual lncRNA signature, the combination of all 7-identified-lncRNA signature showed a much better performance testified by a significantly higher AUC value (AUC = 0.93, 95% CI = 0.86 - 1.00) (Figure 5B).

### Prognostic value of the combined seven-lncRNA signature

According to the risk score, patients in the dataset GSE30161 were further divided into low-score and high-score groups, with the median risk score taken as the cut-off point (Figure 5C). As presented in Figure 5C, our results showed that patients with low risk scores always had a longer progression-free survival (PFS) time. In contrast, the overall survival (OS) time between high- and low- risk groups did not reach a significant difference. In order to confirm whether the seven-lncRNA expression signature was an independent predictor of ovarian patients’ sensitivity to platinum-based paclitaxel chemotherapy, the Kaplan-Meier plotter analysis was conducted. The patients with a high risk score showed a shorter OS time, compared with their counterparts in the low risk group (MST = 40.40 vs 50.03 months). However, the association between seven-lncRNA expression signature and OS did not reach a significant difference (HR = 1.29, 95% CI = 0.63-2.64, *p* = 0.490). Interestingly, when analyzing the association between seven-lncRNA expression signature and PFS, we found that patients with higher risk score had a shorter PFS time (MST = 10.30 vs 15.13 months) with a significant LogRank*p*-value (R = 2.05, 95% CI = 1.20-4.23, *p* = 0.015) (Figure 5D). Together, these results showed that the combined seven-lncRNA signature is an independent predictor of ovarian patients’ sensitivity to platinum-basedpaclitaxel-containing chemotherapy.

### Function annotation of seven-lncRNA signature

**Figure 6:**
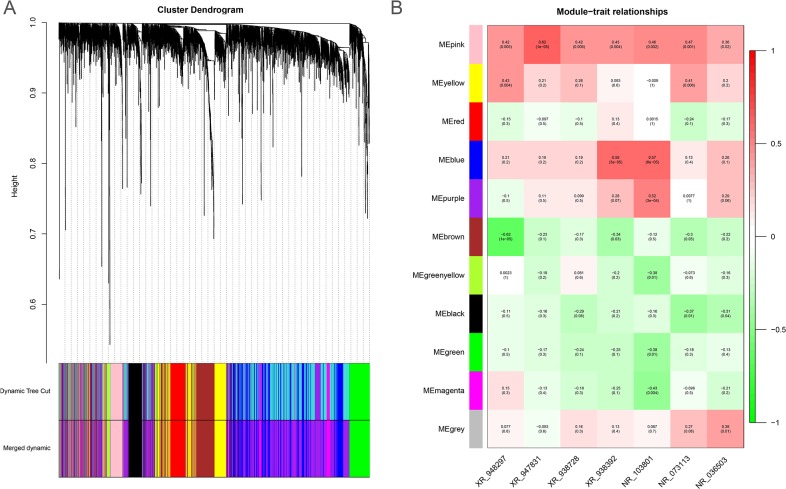
The WGCNA gene-coexpression analysis of differentially expressed genes from the EOC dataset GSE30161 **(A)** The cluster dendrogram differentially expressed mRNAs and lncRNAs derived from the EOC dataset GSE30161. **(B)** The module-trait relationship analysis of the co-expressed patterns between the seven lncRNAs and genome-wide genes. ME: module eigengenes.

**Figure 7:**
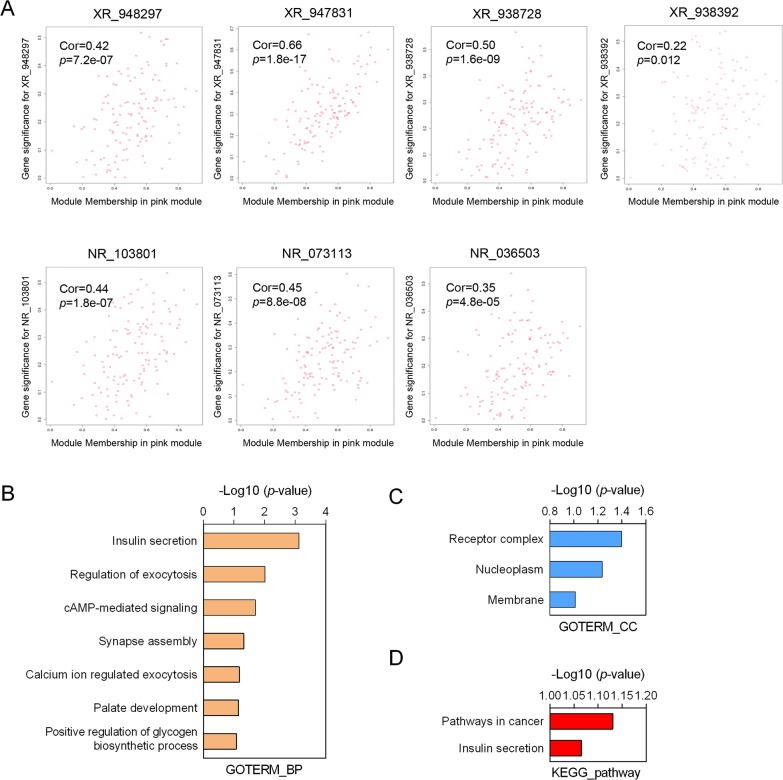
Function annotation of seven-lncRNA signature **(A)** Correlation of each individual lncRNA with the differentially expressed genes from the EOC dataset GSE30161. **(B-C)** Gene Ontology analysis of biological processes (B) and cellular components (C) related to the 129-seven lncRNAs-co-expressed genes. **(D)** The KEGG pathway enrichment analysis of the predicted functions of the seven lncRNAs. GO: Gene Ontology.

To predict the biological function ofthe seven identified lncRNAs, we performed WGCNA based on the mRNA and lncRNA profiles derived from GSE30161 (Figure 6A). The co-expressed relationships between the expression levels of seven lncRNAs and genome-wide genes were investigated by module-trait relationship analysis (Figure 6B), and we identified 129 genes (pink module) with which the expression of seven lncRNAs were all significantly and positively correlated (Figures 6B and 7A). Then we made use of Gene Ontology (GO) analysis and KEGG analysis to analyze the potential biological functions of the 129 picked-up genes. The results revealed these genes are closely involved in biological processes including insulin secretion, regulation of exocytosis, cyclic Adenosine monophosphate (cAMP) -mediated signaling, synapse assembly, calcium ion regulated exocytosis (Figure 7B), and cellular components such as receptor complex, nucleoplasm and membrane (Figure 7C). KEGG pathway enrichment analysis further suggested that those genes were associated with cancer and insulin secretion (Figure 7D). Interestingly, both GO and KEGG pathway enrichment analyses suggested the insulin secretion related pathways were involved in the predicted functions of the sevenlncRNAs. Above findings provide evidence to explore molecular mechanism of paclitaxel resistance in EOC patients.

## DISCUSSION

Most EOC patients respond well at the initial of chemotherapy, but quickly develop drug resistance, which finally leads to treatment failure. To date, there are still no effective methods to prevent drug resistance in EOC. To design new and more effective treatments, it is essential to understand the underlying molecular mechanism inducing drug resistance. In this study, a comprehensive analysis of lncRNA expression profiles in EOC patients was conducted. To the best of our knowledge, our study is the first to explore the association between lncRNAs expression signature and paclitaxel treatment in EOC patients.

So far, although increasing numbers of lncRNAs have been discovered and recorded in biological databases, most of their functions still remain unclear. Previous studies found aberrant lncRNA expressions could indicate the range of tumor progression and have a great power in the diagnosis and prognosis of cancer as novel independent molecular biomarkers [26, 27]. Certain lncRNA, such as HOTAIR, has been identified as the survival biomarker of EOC patients. However, whether lncRNA expression signatures could be used as predictors of paclitaxel chemo-sensitivity in EOC patients have not been determined. Qiao. et al. identified a lncRNA, CCAT1, regulates paclitaxel chemo-sensitivity in nasopharynx cancer cells by manipulating the miR-181a/CPEB2 axis [28]. It is demonstrated that lncRNA H19 is an important element related to paclitaxel chemo-resistance in the ER-positive breast carcinoma cells [29]. In our study, informatics prediction results showed that the genes associated with the seven lncRNAs(XR_948297, XR_947831, XR_938728, XR_938392, NR_103801, NR_073113, and NR_036503) significantly participated in cancer pathways. The predictive capacity of the seven-lncRNA signature in chemotherapeutic sensitivity was identified via ROC analysis. Our findings revealed that the seven-lncRNA signature had a relatively high predictive accuracy of chemoresistance in EOC patients with paclitaxel-containing chemotherapy and was associated with patients’ PFS time. Therefore, based on those findings as well as our analysis, we concluded the seven identified lncRNAsmight be potential biomarkers for chemoresistance in EOC patients. Admittedly, more and further studies are in urgent need to confirm the connection between the seven-lncRNA signature and relevant pathways.

Biologically diverse mechanisms are involved in the development of chemoresistant phenotype in EOC. Among the various biological functions predicted by bioinformatics, insulin and Insulin-like growth factor (IGF) signaling pathways stood out from the crowd to be the topof the list. Actually, insulin and IGF signaling have long been discovered to regulate cellular growth, proliferation, metabolism, and survival [30]. Interestingly, chemo-resistant cells also displayed an enhanced proliferative response to insulin [31]. Insulin and insulin analogs are reported to be associated with a high proliferation rate and chemoresistance in patients with acute lymphoblastic leukemia [32]. Previous study found that high levels of insulin conferred AKT signaling activation and resistance to oxaliplatin in colon cancer cell lines [33]. A recently published research suggests that stroma-derived IGFs can blunt the response to chemotherapy in pancreatic cancer via an IGF-insulin/IGF1R paracrine signaling axis [34]. A recent study also demonstrated that exogenous IGF-I increased cell growth in the ovarian cancer cell line OVCAR-3 in a manner equivalent to 10% FCS [35]. Hyperactivation of the IGF-IR and PI3K signaling pathways has been revealed as an essential event for cisplatin resistance in ovarian cancer cells [36]. Furthermore, PI3KCA is regarded as an oncogene in EOC onset and development, while PIK3R1 is constitutively activated via mutations [37, 38]. Together, these findings indicate a close interaction between chemoresistance and insulin and IGF signaling pathways in EOC.

Considering the regulatory relationship between the seven lncRNAs and prognosis of EOC patients, we further studied the prognostic value of the combination of these seven lncRNAs in EOC patients. As expected,survival analysis confirmed the significant association between the seven-lncRNA signature and EOC patients’ PFS. However, we did not find a significant association between the seven-lncRNA signature and OS. The possible explanation might be that the sevenlncRNAs were identified as paclitaxel-resistance-related biomarkers. Their expression levels could be used to predict chemoresistance but not death. Admittedly, the moderate sample size in our study limited the validity of some stratified analyses to some extent. Although the diagnostic power for these seven lncRNAs in combination is rather modest, our present study offers evidence for the future investigation on whether these seven lncRNAs could be used as prognostic markers in EOC management. Thereby, numerous prospective validation studies need to be performed before they are applied to clinical practice. Besides, another limitation of our present study is the lack of serum levels of those seven lncRNAs. Since blood-based biomarkers are minimally invasive and more important for monitoring therapeutic effects of chemotherapy, We plan to explore the clinical values of serum lncRNAs in the next step.

In summary, we identified seven lncRNAs which were shown to be distinctively expressed in most ovarian tumor samples and significantly correlated with a poor chemotheraputic response of EOC patients. Mechanistically, lncRNA promotes the malignant activities of ovarian cancer cells via cancer-related pathways and insulin secretion. In addition, our study indicates a joint effect of lncRNA-gene markers in predicting the prognosis of EOC patients. Our data also shed light on the potential of the seven lncRNAs as novel therapeutic targets for future treatment of EOC.

## MATERIALS AND METHODS

### Microarray data

The Gene Expression Omnibus (GEO, http://www.ncbi.nlm.nih.gov/geo) is a public database available. Two gene expression profiles (GSE54772 and GSE30161) were obtained from GEO database. GSE54772 consisted of 2 paclitaxel-resistant ovarian cancer cell line samples and 2 paclitaxel-sensitive ovarian cancer cell line samples [21]. GSE30161 included 21 IR (incomplete response) ovarian cancer samples and 25 CR (complete response) ovarian cancer samples [22].

### Weighted correlation network analysis (WGCNA)

A total of 3187probe sets with 2730corresponding differential expressed geneswere annotated to construct a co-expression network using the Rpackage “WGCNA” according to previous reports [23]. The soft thresholding power was selected to 6 to produce a weightednetwork. The enrolled genes were hierarchically clustered based on their topological overlap (TO). Modules of clustered genes were then selected using the Dynamic Tree Cut algorithm. The module eigengenes (ME) were calculated by principal component analysis and clustered according to their correlation to quantify the co-expression similarity of entire modules.

### Receiver operating characteristic analysis

In order to explore the possibility whether lncRNAs could be potential biomarkers for EOC diagnosis and prognosis prediction and assess the risk between two groups of patients, we did receiver operating characteristic (ROC) analysis. The area under the ROC curve (AUC) was used to evaluate the classification performance of the lncRNA signature according to their capability to distinguish IR patients from CR patients.

### Pathway enrichment analysis

Gene ontology analysis (GO) is the most common useful method of annotating genes and gene products and of identifying characteristic biological properties for high-throughput genome or transcriptome data [24]. Kyoto Encyclopedia of Genes and Genomes (KEGG) is a tool about systematic analysis of gene functions, connecting genomic information with higher-order functional information [25]. We used GO analysis and KEGG analysis to identify the biology processes, cellular components and related pathways of the seven candidate lncRNAs-associated genes. *p*<0.05 was considered as statistically significant.

### Survival analysis

Survival analyses for high- and low- risk EOC patients were carried out based on prognostic data derived from GSE30161, respectively. The prognosis survival and progression free survival analysis for each eligible lncRNA and also the combination of all the seven candidate lncRNAs were performed, respectively.
